# Atherogenic index of plasma predicts progression of diabetic kidney disease

**DOI:** 10.3389/fmed.2026.1830550

**Published:** 2026-06-05

**Authors:** Hong-Mou Shih, Sung-Chen Liu, Kuo-Liong Chien, Hsien-Yu Fan, Ming-Chieh Tsai, Shih-Ming Chuang

**Affiliations:** 1 Division of Nephrology, Department of Internal Medicine, MacKay Memorial Hospital, Taipei, Taiwan; 2 School of Medicine, College of Medicine, MacKay Medical University, New Taipei City, Taiwan; 3 Graduate Institute of Physiology, College of Medicine, National Taiwan University, Taipei, Taiwan; 4 Division of Endocrinology and Metabolism, Department of Internal Medicine, MacKay Memorial Hospital, Taipei, Taiwan; 5 Institute of Epidemiology and Preventive Medicine, College of Public Health, National Taiwan University, Taipei, Taiwan; 6 School of Nutrition and Health Sciences, College of Nutrition, Taipei Medical University, Taipei, Taiwan

**Keywords:** albuminuria, atherogenic index of plasma, diabetic kidney disease, dyslipidemia, type 2 diabetes

## Abstract

**Background:**

The atherogenic index of plasma (AIP), calculated as the logarithm of the triglyceride-to-high-density lipoprotein–cholesterol ratio, reflects atherogenic dyslipidemia common in type 2 diabetes (T2D).

**Methods:**

This longitudinal cohort study included 936 adults with type 2 diabetes. Baseline AIP was calculated as log10(triglyceride/high-density lipoprotein cholesterol), and was analyzed continuously and by quartiles. The outcomes were albuminuria progression and sustained ≥50% decline in estimated glomerular filtration rate (eGFR). Associations were assessed using Cox proportional hazards models adjusted for demographic, metabolic, and baseline kidney measures.

**Results:**

Higher AIP was independently associated with greater hazards of kidney outcomes. Each standard-deviation increase in AIP was associated with albuminuria progression (adjusted hazard ratio [HR] 1.25, 95% confidence interval [CI] 1.06–1.47, *P* = 0.007) and sustained eGFR decline (adjusted HR 1.59, 95% CI 1.25–2.02, *P* < 0.001). Compared with the lowest AIP quartile, the highest quartile was associated with higher risks of albuminuria progression (adjusted HR 2.09, 95% CI 1.23–3.57, *P* = 0.007) and eGFR decline (adjusted HR 3.32, 95% CI 1.53–7.24, *P* = 0.002). Associations were directionally consistent across subgroups.

**Conclusion:**

Atherogenic index of plasma may serve as a simple marker of diabetic kidney disease progression risk in T2D, with stronger associations for eGFR loss than for albuminuria progression.

## 1 Introduction

Diabetic kidney disease (DKD) is the most common cause of end stage kidney disease (ESKD) requiring dialysis or transplantation globally and more than half of incident kidney failure cases are attributed to diabetes, underscoring its dominant contribution to kidney failure worldwide (1, 2). DKD is associated with reduced health-related quality of life and higher healthcare utilization and costs, and is strongly linked to cardiovascular morbidity and all-cause mortality (3–5). Therefore, early identification of DKD risk and prompt intervention are essential to improving type 2 diabetes (T2D) outcomes and mitigating the population health burden (6).

Within the KDIGO framework, prognosis is stratified by the joint classification of estimated glomerular filtration rate (eGFR) and albuminuria on a color-graded grid, with each dimension carrying independent prognostic weight (7). In the earliest stages of DKD, increased urinary albumin excretion is often the first detectable abnormality despite preserved eGFR. Accordingly, transitions from A1 (normal to mildly increased albuminuria; urinary albumin-to-creatinine ratio [UACR] < 30 mg/g) to A2 (moderately increased; UACR 30–300 mg/g, formerly “microalbuminuria”) or A3 (severely increased; UACR > 300 mg/g, formerly “macroalbuminuria”), and from A2 to A3, are clinically meaningful because they frequently precede substantial eGFR decline and signal higher risk of progression.

In T2D, atherogenic dyslipidemia, characterized by elevated triglycerides, low high-density lipoprotein (HDL) cholesterol (HDL-C), and enrichment of small dense low-density lipoprotein (LDL), may promote glomerular and tubulointerstitial injury through lipotoxic and inflammatory pathways (8, 9). This lipid phenotype is particularly relevant to DKD because atherogenic lipid abnormalities are also common in CKD, where hypertriglyceridemia and impaired HDL metabolism are accompanied by qualitative changes in HDL particles. In this setting, HDL may have reduced cholesterol transport capacity and diminished antioxidant and anti-inflammatory functions, potentially contributing to atherosclerosis and adverse kidney–cardiovascular outcomes (10). The atherogenic index of plasma (AIP), obtained by log-transforming the triglyceride-to-HDL-C ratio, integrates elevations in triglyceride-rich lipoproteins with reduced HDL-C and serves as a concise indicator of atherogenic dyslipidemia (11). Several studies have shown that elevated AIP is associated with diabetes (12–14). A meta-analysis further reported that the risk of T2D increases with high AIP (15). Among individuals with T2D, higher AIP has also been linked to macrovascular complications, including coronary artery disease and major adverse cardiovascular events (16, 17). Nevertheless, whether baseline AIP independently predicts albuminuria progression and eGFR decline remains uncertain in T2D.

To address this, we conducted a longitudinal cohort study in adults with T2D to determine whether baseline AIP independently predicts albuminuria progression and eGFR decline. We hypothesized that AIP reflects an atherogenic milieu that signals DKD progression risk beyond established factors.

## 2 Materials and methods

### 2.1 Study population and eligibility

Data for this study were obtained from the endocrinology outpatient departments of two branches of MacKay Memorial Hospital in Taiwan, the Taipei branch and the Tamsui branch, through the Diabetes Shared Care Program. This multicenter cohort included adults with type 2 diabetes (aged 20–91 years) who were evaluated between 2018 and 2021 for the assessment of diabetic complications. All participants underwent standardized medical history taking and physical examination, as well as routine laboratory testing approximately every 3 months.

**FIGURE 1:**
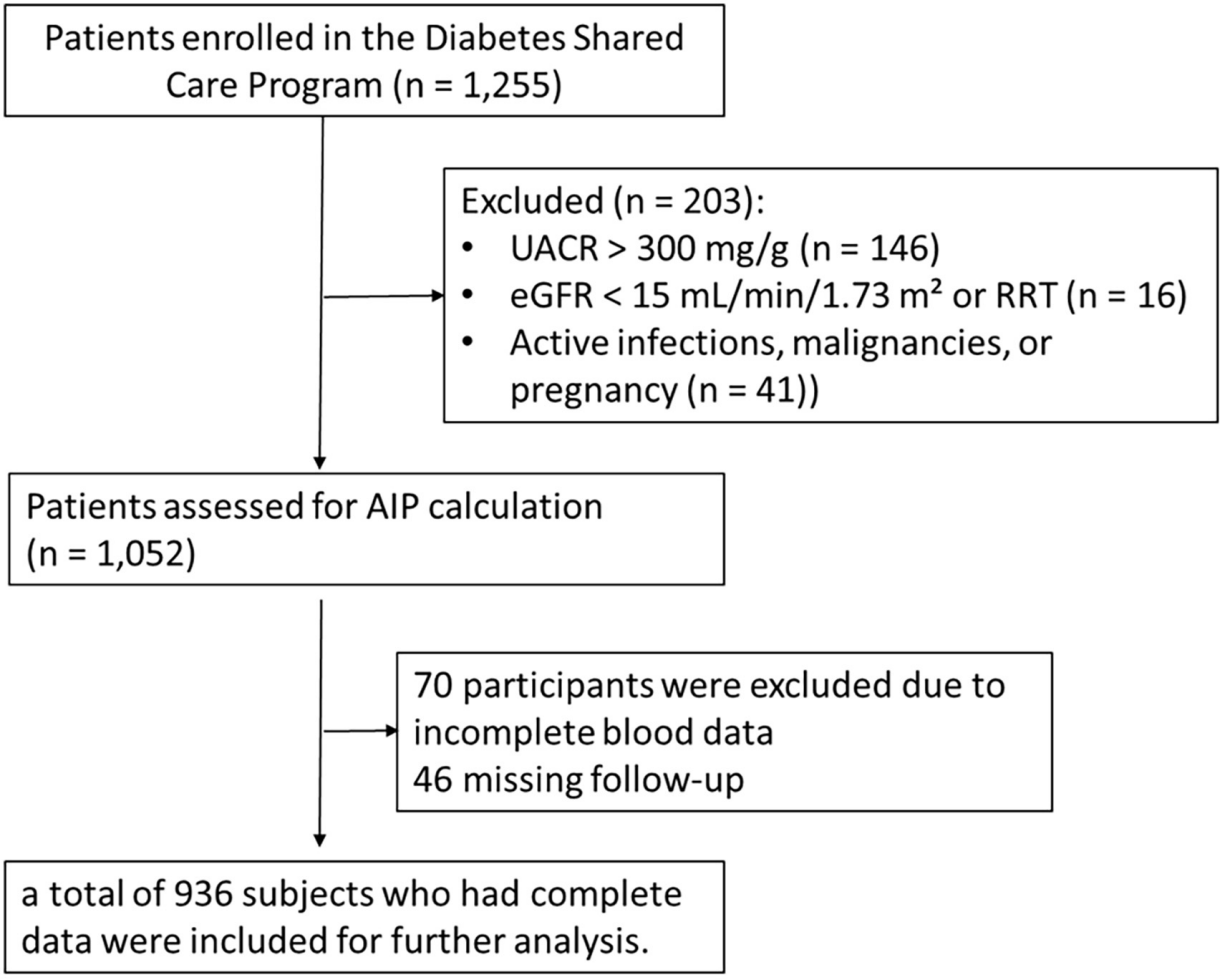
Flow diagram of cohort selection. The diagram shows the number of patients assessed for diabetes complication evaluation, the number meeting the inclusion criteria, and the number excluded for each prespecified reason, resulting in the final analytic cohort of 936 participants.

Eligibility required complete baseline data for serum creatinine, eGFR, UACR, and lipid measures sufficient to calculate the AIP. We excluded individuals with A3 albuminuria (UACR > 300 mg/g), eGFR < 15 mL/min/1.73 m^2^, ongoing kidney replacement therapy, active infectious or malignant disease, or pregnancy. The cohort selection process is summarized in Figure 1. This study protocol was approved by the Institutional Review Board of MacKay Memorial Hospital (IRB number: 18MMHIS104e), and all data access and analyses were conducted in strict accordance with the approved guidelines.

### 2.2 Measurements and definitions

Collected data included age, blood pressure, anthropometrics [waist circumference and body mass index (BMI)], fasting plasma glucose (FPG), glycated hemoglobin (HbA1c), lipid profile, liver enzymes, serum creatinine (Scr), eGFR, and UACR. eGFR was estimated using the CKD-EPI 2021 creatinine equation (race-free): 142 × min(Scr/κ, 1)^∧^α × max(Scr/κ, 1)^∧^−1.200 × 0.9938^∧^Age × [1.012 if female], where Scr is serum creatinine (mg/dL); κ = 0.7 for females and 0.9 for males; α = −0.241 for females and −0.302 for males. Albuminuria categories followed KDIGO: A1 (<30 mg/g), A2 (30–300 mg/g), and A3 (≥300 mg/g). Atherogenic index of plasma (AIP) was the primary exposure and was analyzed both continuously and by quartiles. AIP was calculated as the base-10 logarithm of the triglyceride-to-HDL-C ratio using values in mmol/L (log10[Triglyceride/HDL-C]). For descriptive and categorical analyses, AIP was grouped as Q1 < −0.17, Q2 −0.17 to <0.03, Q3 0.03 to <0.22, and Q4 ≥ 0.22.

### 2.3 Outcome variables

We evaluated two kidney outcomes, including albuminuria progression and eGFR decline. Albuminuria progression was defined as transition from KDIGO A1 to A2/A3 or from A2 to A3, confirmed by at least two consecutive UACR measurements and more than 1 year of follow-up to minimize misclassification. eGFR decline was defined as a ≥ 50% reduction in eGFR from baseline, sustained on repeat testing during follow-up. For each participant, at least one measurement of serum creatinine or eGFR was obtained annually and compared with baseline values recorded at study entry.

### 2.4 Follow-up and censoring

The follow-up period for each participant commenced on the date of enrollment in the Diabetes Shared Care Program after completion of baseline data collection. For each outcome-specific time-to-event analysis, follow-up ended at the earliest occurrence of the corresponding outcome, the last recorded clinical visit, or the end of the study period. Participants without the corresponding outcome were censored at the end of follow-up or at the last recorded visit if they were lost to follow-up, defined as more than 12 months without a clinical visit. The event status was coded as 1 for participants experiencing the corresponding outcome, including albuminuria progression or sustained ≥50% eGFR decline, and 0 for censored observations.

### 2.5 Statistical analysis

Baseline characteristics across AIP quartiles were summarized as mean ± standard deviation (SD) or median (interquartile range), as appropriate according to their distribution. Group differences were evaluated using the ANOVA or Wilcoxon rank-sum test for continuous variables, and the chi-square test for categorical variables presented as counts and percentages. The Kaplan–Meier methods with log-rank tests were applied to estimate the cumulative incidence of the primary outcomes.

Crude event rates were calculated as the number of events divided by total person-years of follow-up and expressed per 1,000 person-years. Follow-up duration was summarized as the median across AIP quartiles. We performed univariate and multivariate Cox proportional hazards analyses to assess the risk of adverse outcomes. In the multivariate analysis, the model was adjusted for the following potential confounding factors: age, sex, BMI, hypertension, LDL-C, HbA1c, eGFR, and baseline UACR. These confounders were pre-specified based on clinical relevance and prior literature. Multicollinearity was assessed using Variance Inflation Factors (VIF), with all values <5 indicating no significant collinearity. Detailed VIF values are provided in Supplementary Table 1. AIP was modeled as a continuous predictor using restricted cubic splines within Cox proportional hazards regression to examine its association with the hazards of albuminuria progression and eGFR decline. Subgroup analyses were performed to evaluate whether the associations between AIP and kidney outcomes differed by sex, age category, metabolic syndrome status, CKD status, baseline albuminuria category, and baseline eGFR category. Baseline albuminuria was categorized as A1 (<30 mg/g) or A2 (30– < 300 mg/g), and baseline eGFR was categorized as <45, 45–60, or >60 mL/min/1.73 m^2^. Effect modification was assessed by adding multiplicative interaction terms between AIP and each subgroup variable to the Cox proportional hazards models. Results are presented as hazard ratios (HR) with 95% confidence intervals (CI), together with *P*-values for interaction.

## 3 Results

A total of 936 participants were enrolled in this study. Table 1 shows the baseline characteristics across quartiles of AIP. Significant differences were observed in age (*P* = 0.004) and BMI (*P* < 0.001) among the four groups. Metabolic factors, including triglycerides and HDL-C, varied markedly across AIP categories (both *P* < 0.001), reflecting the components of the AIP calculation. The prevalence of hypertension and hyperlipidemia also increased progressively with higher AIP levels (*P* = 0.003 and *P* = 0.006, respectively). Kidney function indicators demonstrated significant group differences, with creatinine (*P* = 0.003) and eGFR (*P* = 0.005) showing trends across the quartiles. In contrast, HbA1c, liver enzymes, uric acid, and albuminuria did not differ significantly between groups. During a mean follow-up of 38 months, the composite outcome of kidney failure (incident renal replacement therapy and eGFR < 15 mL/min/1.73 m^2^) occurred in 16 patients (1.71%). Due to the limited number of events, these findings are reported descriptively rather than through formal survival analysis.

**TABLE 1:** Baseline characteristics by atherogenic index of plasma quartiles.

Characteristics	Total	Q1 (<−0.17)	Q2 (−0.17 to 0.03)	Q3 (0.03–0.22)	Q4 (≥0.22)	*P*
*N*	936	235	234	234	233	
Age (year)	64.9 ± 10.0	66.1 ± 9.3	66.0 ± 9.66	64.5 ± 10.1	63.2 ± 10.7	0.004
Sex (male, %)	44	42	41	46	48	0.311
Smoking (%)	12	11	8	15	14	0.156
BMI (kg/m^2^)	25.8 ± 4.2	24.2 ± 3.7	26.0 ± 4.3	26.3 ± 4.2	26.9 ± 4.2	<0.001
DM duration (years)	11.0 ± 7.7	11.6 ± 7.4	11.3 ± 7.6	10.9 ± 8.7	10.2 ± 6.9	0.071
Hypertension (%)	64	56	63	65	72	0.003
Hyperlipidemia (%)	72	64	74	71	78	0.006
FPG (mmol/L)	8.3 ± 2.8	8.0 ± 2.4	7.9 ± 2.3	8.2 ± 2.6	8.9 ± 3.7	<0.001
HbA1c (%)	7.7 ± 3.6	7.4 ± 1.4	7.9 ± 6.8	7.5 ± 1.4	8.0 ± 1.9	0.209
TC (mmol/L)	4.5 ± 1.0	4.5 ± 0.8	4.4 ± 0.9	4.5 ± 1.0	4.7 ± 1.1	0.087
TG (mmol/L)	1.5 ± 1.1	0.7 ± 0.3	1.1 ± 0.3	1.5 ± 0.4	2.7 ± 1.6	<0.001
LDL-C (mmol/L)	2.5 ± 0.7	2.5 ± 0.7	2.6 ± 0.8	2.7 ± 0.8	2.5 ± 0.8	0.025
HDL-C (mmol/L)	1.3 ± 0.4	1.6 ± 0.4	1.3 ± 0.2	1.1 ± 0.2	0.9 ± 0.2	<0.001
UA (mmol/L)	0.4 ± 0.1	0.3 ± 0.1	0.4 ± 0.1	0.4 ± 0.1	0.4 ± 0.1	0.196
SBP (mmHg)	143.1 ± 19.6	141.00 ± 19.74	143.66 ± 18.83	142.63 ± 20.30	145.39 ± 19.26	0.093
DBP (mmHg)	78.7 ± 10.4	77.00 ± 10.11	78.64 ± 9.84	78.81 ± 11.63	80.66 ± 9.87	0.002
GPT (U/L)	26.7 ± 18.6	26.13 ± 26.14	26.17 ± 15.39	26.29 ± 14.46	28.31 ± 14.21	0.57
Cr (mg/dL)	1.0 ± 0.6	0.88 ± 0.52	0.98 ± 0.49	0.97 ± 0.54	1.07 ± 0.65	0.003
eGFR (mL/min/1.73 m^2^)	79.9 ± 30.7	85.04 ± 27.50	79.53 ± 34.49	79.54 ± 28.88	75.04 ± 31.24	0.005
UACR (mg/g)	33.0 ± 53.1	25.6 ± 44.1	35.8 ± 51.5	32.1 ± 50.6	43.5 ± 60.6	0.169
A1 albuminuria (<30 mg/g), (%)	72.6	77.3	70.1	74.1	61.8	0.017
A2 albuminuria (30–300 mg/g) (%)	27.3	22.7	29.9	25.9	37.6	0.017
AIP	0.1 ± 0.3	−0.37 ± 0.16	−0.06 ± 0.06	0.11 ± 0.06	0.42 ± 0.22	<0.001

Data are presented as mean value ± standard deviation or %. Q, quartile; BMI, body mass index; DM, diabetes mellitus; FPG, fasting plasma glucose; HbA1c, glycosylated hemoglobin; TC, total cholesterol; TG, triglyceride; LDL-C, low-density lipoprotein cholesterol; HDL-C, high-density lipoprotein cholesterol; UA, uric acid; SBP, systolic blood pressure; DBP, diastolic blood pressure; GPT, glutamic-pyruvic transaminase; Cr, creatinine; eGFR, estimated glomerular filtration rate; UACR, urinary albumin-to-creatinine ratio; AIP, atherogenic index of plasma.

Median follow-up duration was similar across AIP quartiles, ranging from 35.7 to 36.7 months. Crude event rates for both albuminuria progression and sustained ≥50% eGFR decline increased across higher AIP quartiles. For albuminuria progression, the crude event rate increased from 10.4 per 1,000 person-years in Q1 to 48.0 per 1,000 person-years in Q4. For sustained eGFR decline, the corresponding rates increased from 35.2 to 70.6 per 1,000 person-years. Quartile-specific event rates and follow-up durations are summarized in Supplementary Table 2.

Table 2 shows the association between AIP and kidney outcomes. Each standard deviation increase in AIP was linked to a higher risk of albuminuria progression (adjusted HR 1.25, 95% CI 1.06–1.47; *P* = 0.007) and eGFR decline (adjusted HR 1.59, 95% CI 1.25–2.02; *P* < 0.001). Compared with the lowest AIP quartile, the highest quartile was associated with higher risks of albuminuria progression (adjusted HR 2.09, 95% CI 1.23–3.57; *P* = 0.007) and sustained eGFR decline (adjusted HR 3.32, 95% CI 1.53–7.24; *P* = 0.002). A stepwise rise in risk was observed across quartiles, although some intermediate groups did not reach statistical significance.

**TABLE 2:** Risk of albuminuria progression or eGFR decline across quartiles of the atherogenic index of plasma in patients with diabetes.

	Albuminuria progression				eGFR decline			
	Crude		Adjusted		Crude		Adjusted	
Variables	HR (95% CI)	*P*-value	HR (95% CI)	*P*-value	HR (95% CI)	*P*-value	HR (95% CI)	*P*-value
Per SD increment of AIP	1.2 6 (1.08–1.46)	0.003	1.25 (1.06–1.47)	0.007	1.55 (1.26–1.92)	<0.001	1.59 (1.25–2.02)	<0.001
Quartile of AIP								
Q1 (<−0.17)	1	Ref.	1	Ref.	1	Ref.	1	Ref.
Q2 (−0.17 to 0.03)	1.68 (1.01–2.83)	0.049	1.61 (0.93–2.76)	0.088	1.70 (0.73–3.98)	0.220	1.01 (0.40–2.58)	0.972
Q3 (0.03–0.22)	1.95 (1.18–3.23)	0.010	1.80 (1.06–3.07)	0.029	2.77 (1.27–6.04)	0.011	1.98 (0.87–4.55)	0.104
Q4 (≥0.22)	2.12 (1.29–3.48)	0.003	2.09 (1.23–3.57)	0.007	3.90 (1.86–8.17)	<0.001	3.32 (1.53–7.24)	0.002

eGFR, estimated glomerular filtration rate; AIP, atherogenic index of plasma; HR, hazard ratio; SD, standard deviation; Q, quartile. Adjusted for age, sex, BMI, hypertension, LDL-C, HbA1c, eGFR, urinary albumin-to-creatinine ratio.

Baseline missingness is summarized in Supplementary Table 3. In sensitivity analyses using multiple imputation, the associations between AIP and both kidney outcomes remained consistent with the primary complete-case analyses. Each standard-deviation increment in AIP was associated with albuminuria progression and sustained ≥50% eGFR decline, and the highest AIP quartile remained significantly associated with both outcomes (Supplementary Table 4).

**FIGURE 2:**
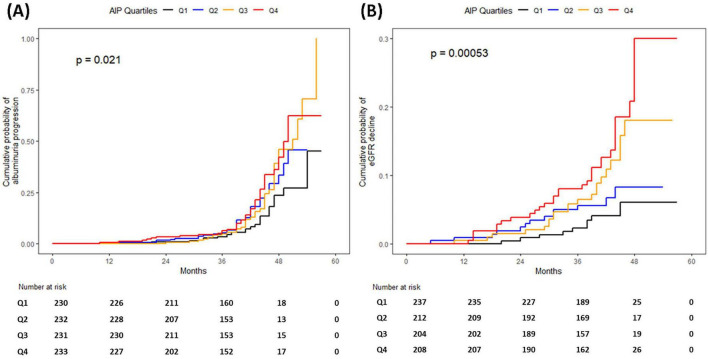
Cumulative probability of albuminuria progression **(A)** or eGFR decline **(B)** across quartiles of the atherogenic index of plasma in patients with diabetes. Number-at-risk tables are shown beneath each panel at key follow-up time points.

Kaplan–Meier analysis showed that higher AIP quartiles were associated with a greater risk of renal deterioration. As shown in Figure 2A, the cumulative probability of albuminuria progression increased across AIP quartiles (log-rank *P* = 0.0212). Similarly, in Figure 2B, patients in higher AIP quartiles had a higher probability of eGFR decline, with a significant difference among groups (log-rank *P* < 0.001).

**FIGURE 3:**
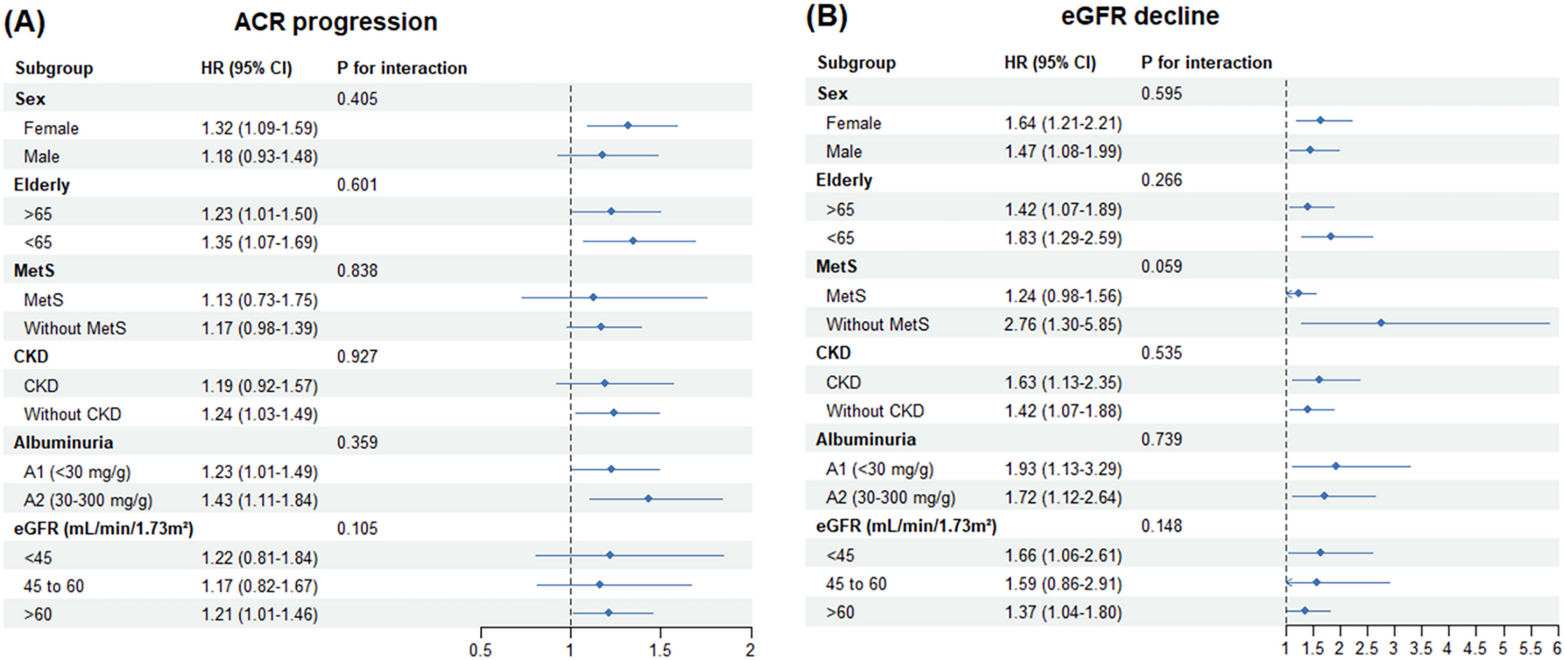
Forest plots of subgroup analyses and interaction effects showing HRs and 95% CIs for the associations between AIP and **(A)** albuminuria progression and **(B)** eGFR decline. AIP, atherogenic index of plasma; eGFR, estimated glomerular filtration rate; HR, hazard ratio; CI, confidence interval; MetS, metabolic syndrome; CKD, chronic kidney disease.

In the subgroup analyses presented in Figure 3, the associations of AIP with albuminuria progression and eGFR decline were generally directionally consistent across strata defined by sex, age, metabolic syndrome, and chronic kidney disease (CKD) status, baseline albuminuria category, and baseline eGFR category. All subgroups showed an increased risk, and none of the interaction tests reached statistical significance, indicating an absence of appreciable effect modification across these groups.

**FIGURE 4:**
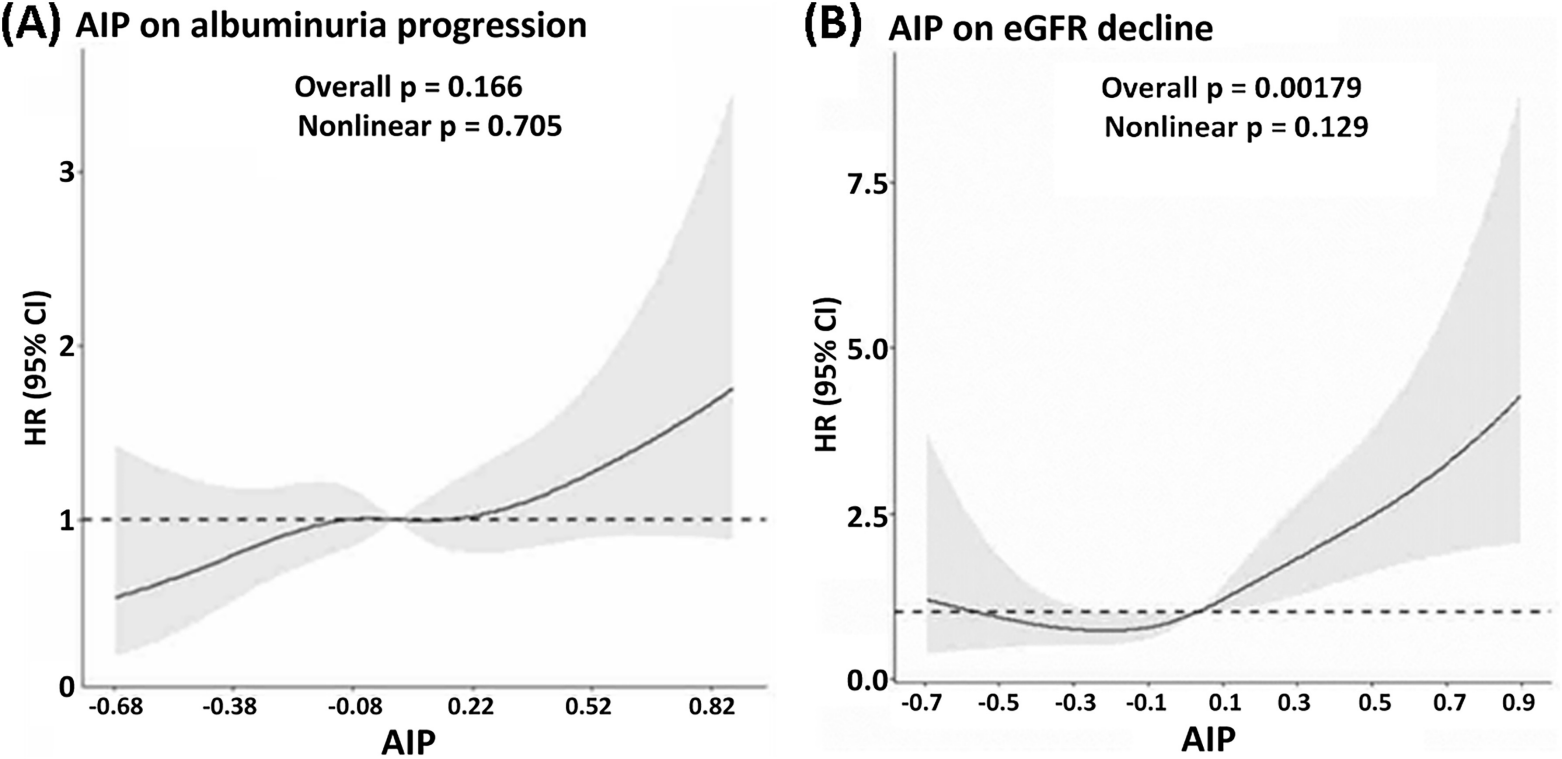
Restricted cubic splines for the association between AIP and **(A)** albuminuria progression and **(B)** eGFR decline. AIP, atherogenic index of plasma; eGFR, estimated glomerular filtration rate; HR, hazard ratio; CI, confidence interval.

Figure 4 illustrated the restricted cubic spline analysis examining the association between AIP and kidney outcomes. The overall association between AIP and albuminuria progression was not statistically significant (overall *p* = 0.166; nonlinear *p* = 0.705). Nonetheless, the spline curve indicated that higher AIP levels were accompanied by an increased risk of albuminuria progression compared with lower AIP levels. For eGFR decline, a significant overall association with AIP was observed (overall *p* < 0.001), and the risk rose steadily with increasing AIP. No significant nonlinear pattern was identified (nonlinear *p* = 0.129).

## 4 Discussion

In our longitudinal cohort of adults with T2D, higher baseline AIP was independently associated with eGFR decline, showing an approximately linear exposure–response in restricted cubic-spline analyses and clear separation of cumulative-incidence curves across AIP quartiles. Quartile models also demonstrated graded increases in the risk of albuminuria progression, although the spline-based global test for albuminuria did not reach statistical significance. These patterns were broadly consistent across subgroups and remained robust across multiple sensitivity analyses. Notably, the threshold defining the highest AIP quartile in our cohort (≥0.22) closely corresponds to previously proposed AIP ranges associated with increased cardiovascular risk, commonly defined as values above approximately 0.21. This concordance supports the clinical interpretability of the observed kidney-risk gradient, although the present quartile boundary was data-derived and should not be regarded as a kidney-specific diagnostic threshold (18).

From a mechanism standpoint, atherogenicity reflects the propensity of circulating lipoproteins to deliver and retain cholesterol within the arterial wall. Triglyceride-rich lipoproteins and their remnants are now considered causal drivers of atherosclerotic disease, while small, dense LDL particles exhibit greater arterial wall penetration, oxidative susceptibility, and longer residence time, thereby heightening their atherogenic potential (19, 20). HDL-C is considered anti-atherogenic because it participates in reverse cholesterol transport, facilitating cholesterol efflux from arterial wall macrophages and its subsequent hepatic clearance. Low HDL-C has repeatedly been linked to higher cardiovascular risk in observational cohorts, and triglyceride-rich remnant particles are now recognized as direct contributors to atherosclerotic plaque formation (21). Within the metabolic context of T2D, AIP integrates these dimensions by capturing a triglyceride-rich, low-HDL lipid profile that correlates with an excess of small, dense LDL and triglyceride-rich remnant lipoproteins. These features denote increased atherogenic potential and diminished cholesterol transport capacity, offering a compact surrogate of atherogenic dyslipidemia that may be relevant not only to vascular disease but also to kidney disease progression (22–24).

In the CKD setting, lipid-related risk is not fully captured by conventional lipid concentrations alone. Although LDL-lowering with statin-based therapy remains the cornerstone of lipid management, CKD is accompanied by qualitative HDL remodeling in the uremic and inflammatory milieu (10). These changes may impair reverse cholesterol transport and attenuate the anti-inflammatory, antioxidant, and antithrombotic properties of HDL, thereby weakening its atheroprotective effects and promoting endothelial dysfunction and atherosclerosis. In parallel, hypertriglyceridemia and triglyceride-rich remnant lipoproteins may contribute to atherosclerosis through arterial-wall cholesterol delivery, oxidative stress, and inflammation (25). Therefore, AIP may be clinically relevant not simply as a triglyceride-to-HDL-C ratio, but as an integrated marker of a triglyceride-rich, low-HDL, and functionally adverse lipid milieu that is biologically plausible in CKD and diabetic kidney disease.

Within the kidney, diabetes perturbs lipid handling along both influx and disposal pathways. In proximal tubules, fatty-acid entry increases via basolateral CD36/fatty acid translocase and apical uptake of albumin-bound fatty acids (26, 27). Hyperglycemia suppresses AMPK activation and, consequently, dampens PPARα/PGC-1α–driven transcription, limiting CPT-1–mediated mitochondrial import of long-chain fatty acids and blunting β-oxidation (28). High glucose also downregulates the cholesterol transporter, ATP-binding cassette transporter A1 (ABCA1), thereby impairing cholesterol efflux and promoting intracellular lipid accumulation (29). These alterations favor intrarenal triglycerides and cholesterol accumulation with downstream lipotoxic signaling and have been documented in experimental models and human DKD biopsies (30). The resulting lipid burden promotes endothelial dysfunction and oxidative stress, and lipotoxic injury in podocytes and proximal tubules, fostering glomerulosclerosis and tubulointerstitial fibrosis (31). In addition, higher AIP correlates with insulin resistance and with steatotic liver disease, metabolic states that may further amplify kidney inflammation and hemodynamic stress (32). Collectively, this biology plausibly links an atherogenic lipid profile to progressive loss of filtration even when albuminuria changes are modest.

Several studies have examined the association between the AIP and kidney outcomes in T2D; however, most are cross-sectional (33–39), and only a limited number are longitudinal. In a prospective T2D cohort, Zhang et al. reported that higher baseline AIP and unfavorable AIP trajectories were associated with incident DKD, defined by crossing eGFR < 60 or UACR ≥ 30 mg/g, rather than by within-category albuminuria progression or percentage eGFR decline (40). In a mixed metabolic cohort, Oh et al. showed that the highest AIP quartile predicted an eGFR-based composite (incident eGFR < 60, ≥30% eGFR decline, or initiation of kidney replacement therapy), with no albuminuria data available (41). In community-dwelling adults, Liu et al. linked AIP to rapid eGFR decline and incident CKD; albuminuria endpoints were also not included and the sample was not restricted to T2D (42). Against this background, our studies extend prior literature by demonstrating prognostic value for clinically meaningful kidney endpoints in diabetes. Importantly, we evaluated both trajectories of kidney injury, KDIGO-defined albuminuria category transitions and a hard eGFR endpoint in adults with T2D, providing a more granular characterization of kidney disease progression.

The discrepancy between the stronger association of AIP with eGFR decline and the weaker signal for albuminuria transitions is consistent with contemporary reviews emphasizing heterogeneity in DKD trajectories, including non-albuminuric progression (43). Albuminuria is variable and sensitive to short-term influences, such as transient hyperglycemia, blood-pressure fluctuations, and the use or intensification of RAAS blockade or SGLT2 inhibitors, and guideline statements therefore recommend confirmation with repeated UACR measurements to minimize misclassification (1). Moreover, DKD can progress through tubulointerstitial pathways with little change in albuminuria, making eGFR-based endpoints more responsive to systemic metabolic stress captured by AIP (43, 44). Finally, from a modeling standpoint, because data were sparse at the extremes of AIP, spline smoothing likely reduced power for the overall and nonlinearity tests, even though quartile analyses showed a clear risk gradient (45).

This study’s strengths encompass longitudinal follow-up with time-to-event modeling, KDIGO-concordant kidney endpoints, and reproducible associations across subgroups and sensitivity analyses in a diabetic-only cohort. Limitations include potential residual confounding from unmeasured lifestyle factors, such as diet and physical activity, and from differences in lipid-lowering or renoprotective therapies. In particular, because the cohort was enrolled between 2018 and 2021, evolving use of SGLT2 inhibitors and GLP-1 receptor agonists may have influenced kidney outcomes; detailed time-updated information on these agents was not consistently available and could not be fully accounted for in the present analysis. Additional limitations include reliance on a single baseline AIP measurement, possible misclassification from albuminuria variability, and limited power at extreme AIP ranges.

In summary, baseline AIP captures an atherogenic milieu relevant to subsequent kidney function loss in type 2 diabetes and shows weaker, less certain associations with albuminuria progression. Given that AIP can be derived from routine lipid profiles, our findings suggest that it may provide a simple and practical marker for kidney disease progression risk stratification in real-world care for patients with type 2 diabetes. Future investigations in larger and more diverse populations are warranted to validate its prognostic value and clinical utility. Serial measurements and mediation analyses incorporating apolipoprotein B, remnant cholesterol, and treatment exposures (e.g., SGLT2 inhibitors, GLP-1 receptor agonists, fibrates) should clarify causal pathways and inform whether AIP trajectories improve kidney outcome prediction in diabetes.

## Data Availability

The raw data supporting the conclusions of this article will be made available by the authors, without undue reservation.
