# Supplementary material for: Atherogenic index of plasma predicts progression of diabetic kidney disease
Source: Front Med (Lausanne). 2026 Jun 5;13:1830550. doi: 10.3389/fmed.2026.1830550 (PMC13278895; doi:10.3389/fmed.2026.1830550)
Supplement: Supplementary file 1 [file Table_1.docx]

Supplementary Material

# Supplementary Tables

| **Supplementary Table S1. Variance inflation factor (VIF) for collinearity diagnostics across different models.** | | |
| --- | --- | --- |
| Variable | Model 1: Albuminuria progression (VIF) | Model 2: eGFR decline (VIF) |
| Atherogenic Index of Plasma (AIP) | 1.1 | 1.1 |
| Age | 1.34 | 1.34 |
| Sex | 1.04 | 1.04 |
| BMI | 1.14 | 1.14 |
| Hypertension | 1.1 | 1.1 |
| LDL-C | 1.02 | 1.02 |
| HbA1c | 1.01 | 1.01 |
| eGFR | 1.35 | 1.36 |
| UACR | 1.07 | 1.08 |

Abbreviation: VIF, variance inflation factor; BMI, body mass index; LDL-C, low-density lipoprotein cholesterol; HbA1c, glycosylated hemoglobin; eGFR, estimated glomerular filtration rate; UACR, urinary albumin-to-creatinine ratio.

| **Supplementary Table S2. Crude event rates and follow-up duration stratified by atherogenic index of plasma quartiles** | | | | |
| --- | --- | --- | --- | --- |
|  | **Q1(<-0.17)** | **Q2(-0.17- 0.03)** | **Q3(0.03-0.22)** | **Q4(≥0.22)** |
| Albuminuria progression, events per 1,000 person-years | 10.4 | 20.5 | 30.9 | 48.0 |
| Sustained ≥50% eGFR decline, events per 1,000 person-years | 35.2 | 54.7 | 61.2 | 70.6 |
| Median follow-up (months) | 36.7 | 35.7 | 36.4 | 36.0 |

Abbreviation: eGFR, estimated glomerular filtration rate; Q, quartile.

| **Supplementary Table S3. Missingness in baseline clinical and laboratory variables, n (%)** | | | |
| --- | --- | --- | --- |
| Variable | Missing_N | Total_N | Missing (%) |
| age | 0 | 936 | 0 |
| sex | 0 | 936 | 0 |
| Smoking | 162 | 936 | 17.31 |
| BMI | 0 | 936 | 0 |
| DM duration | 43 | 936 | 4.59 |
| Hypertension | 2 | 936 | 0.21 |
| Hyperlipidemia | 0 | 936 | 0 |
| FPG | 1 | 936 | 0.11 |
| HbA1c | 10 | 936 | 1.07 |
| TC | 19 | 936 | 2.03 |
| TG | 29 | 936 | 3.1 |
| LDL-C | 17 | 936 | 1.82 |
| HDL-C | 57 | 936 | 6.09 |
| UA | 741 | 936 | 79.17 |
| SBP | 1 | 936 | 0.11 |
| DBP | 1 | 936 | 0.11 |
| GPT | 110 | 936 | 11.75 |

Abbreviation: BMI, body mass index; DM, diabetes mellitus; FPG, fasting plasma glucose; HbA1c, glycosylated hemoglobin; TC, total cholesterol; TG, triglyceride; LDL-C, low-density lipoprotein cholesterol; HDL-C, high-density lipoprotein cholesterol; UA, uric acid; SBP, systolic blood pressure; DBP, diastolic blood pressure; GPT, glutamic-pyruvic transaminase.

| Supplementary Table S4. Sensitivity analysis using multiple imputation for predictors of kidney outcomes (Cox proportional hazards model) | | | | |
| --- | --- | --- | --- | --- |
|  | Model 1: Albuminuria progression | | Model 2: eGFR decline | |
| Variable | Adjusted HR (95% CI) | p-value | Adjusted HR (95% CI) | p-value |
| Per SD increment of AIP | 1.27 (1.07–1.50) | 0.009 | 1.59 (1.26–2.00) | <0.001 |
| Quartile of AIP |  |  |  |  |
| Q1(<-0.17) | 1 | Ref. | 1 | Ref. |
| Q2(-0.17- 0.03) | 1.68 (1.03–2.76) | 0.040 | 1.36 (0.56–3.29) | 0.501 |
| Q3(0.03-0.22) | 1.87 (1.08–3.23) | 0.029 | 2.47 (1.09–5.58) | 0.033 |
| Q4(≥0.22) | 2.25 (1.32–3.83) | 0.004 | 3.75 (1.76–8.00) | 0.001 |

Abbreviation: eGFR, estimated glomerular filtration rate; AIP, atherogenic index of plasma; HR, hazard ratio; SD, standard deviation; Q, quartile

Adjusted for age, sex, BMI, hypertension, LDL-C, HbA1c, eGFR, urinary albumin-to-creatinine ratio

**
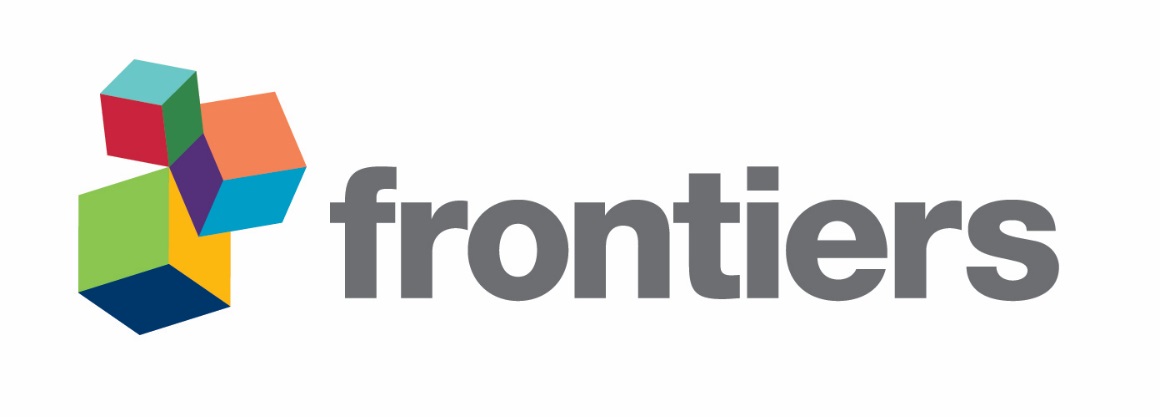
**
